# Preparation of Eggshell–Sodium Alginate/Polyvinyl Alcohol Composite Hydrogel Carrier-Embedded *Rhizobacteria* and Their Storage Properties

**DOI:** 10.3390/gels12080708

**Published:** 2026-08-10

**Authors:** Yanjun Cui, Yongsheng Xiang, Libo Jiang, Shuxia Fang, Aolei He, Tuo Yao, Bing Hu, Jia Wei

**Affiliations:** 1 Key Laboratory of Grassland Ecosystem, Ministry of Education (Gansu Agricultural University), Lanzhou 730070, China; heal@gsau.edu.cn (A.H.); yaotuo@gsau.edu.cn (T.Y.); 2 Institute of Agricultural Resources Chemistry and Application, College of Science, Gansu Agricultural University, Lanzhou 730070, China; 1073323120445@st.gsau.edu.cn (L.J.); 1073324120473@st.gsau.edu.cn (S.F.); bb810410@aliyun.com (B.H.); weij@gsau.edu.cn (J.W.); 3 Lanzhou Petrochemical Research Center, Petrochemical Research Institute, PetroChina, Lanzhou 730060, China

**Keywords:** eggshell powder, composite hydrogel carrier, *rhizobial* inoculant, biodegradable

## Abstract

*Rhizobial* inoculants play a critical role in sustainable agriculture by enhancing biological nitrogen fixation; however, maintaining *rhizobial* viability during storage remains a formidable challenge. In this paper, six embedded *rhizobial* formulations, designated E-SA/PVA R1 through E-SA/PVA R6, were prepared via an embedding method using sodium alginate (SA), polyvinyl alcohol (PVA), and eggshell powder as primary materials with varying component ratios. Structural characterization confirmed the suitability of the composite hydrogel carriers for *rhizobial* encapsulation: Fourier-transform infrared (FTIR) spectroscopy and thermogravimetric analysis (TGA) revealed effective crosslinking between SA and PVA, yielding a stable matrix. Scanning electron microscopy (SEM) confirmed the successful encapsulation of abundant *rhizobia* within the carriers. Mercury intrusion porosimetry (MIP) was employed to characterize the pore architecture of the composite carriers, revealing the relationships among porosity, average pore size, and permeability. Mass transfer evaluation using three probe molecules of differing molecular weights yielded diffusion permeability coefficients (DPC) that quantitatively elucidated the size-dependent transport behavior within the carrier matrices. All embedded formulations displayed favorable biodegradability in soil environments. Notably, the embedded agents maintained viable cell counts above 8 log CFU/g across all formulations even after 60 days under various pH and temperature conditions, satisfying the microbial fertilizer standard in China, establishing them as promising candidates for practical *rhizobial* inoculant applications.

## 1. Introduction

To meet the escalating food demand driven by population growth, modern agriculture has extensively relied on the application of agrochemicals, such as chemical fertilizers and pesticides, to sustain high crop yields [1,2]. However, the long-term and intensive use of these inputs has triggered a series of ecological and environmental problems. The excessive application of chemical fertilizers not only leads to soil compaction and fertility degradation, but also causes water contamination and air pollution [3,4]. This intensive agricultural model has created a vicious cycle that both undermines the sustainable productivity of cultivated land and exacerbates ecological risks and public health hazards. To achieve the goal of green and sustainable agricultural development while safeguarding human health, reducing fertilizer use while enhancing its efficiency, along with the research, development, and application of novel green fertilizers, has become an urgent priority [5,6]. Therefore, microbial fertilizers, which possess both environmentally friendly characteristics and the potential to enhance productivity, have progressively emerged as a research hotspot.

Microorganisms are a vital natural resource for green development [7]. The use of biofertilizers enriched with beneficial microorganisms to improve soil health and enhance crop growth is crucial for balancing high-efficiency production with agricultural sustainability [8]. Studies have demonstrated that microbial fertilizers derived from plant growth-promoting *rhizobacteria* (PGPR) play an important role in improving soil fertility and the promotion of crop growth [9]. As a class of plant probiotics, PGPR contribute to the maintenance of soil fertility and promoting plant development. PGPR that have been extensively studied both domestically and internationally include species from Pseudomonas, Bacillus, Azotobacter, Enterobacter, and Serratia. These bacteria can decompose organic matter, suppress soil-borne diseases, and promote crop growth and resistance [10,11]. Certain strains also possess functions such as biological nitrogen fixation, phosphorus solubilization, potassium solubilization, and phytohormone regulation. Among these, *rhizobia* are a group of soil-dwelling bacteria that form symbiotic associations with leguminous plants, facilitating biological nitrogen fixation through the establishment of root nodules [12]. *Rhizobial* inoculants, preparations containing viable *rhizobial* cells, have been widely adopted to enhance crop productivity and soil fertility across diverse agricultural systems [13,14,15]. Despite their agronomic significance, the practical application of *rhizobial* inoculants is severely constrained by the limited survival of *rhizobial* cells during storage and transportation. Free *rhizobia* are highly susceptible to adverse environmental conditions, including elevated temperatures, extreme pH values, and desiccation, which can rapidly diminish their viability and, consequently, their nitrogen-fixing efficacy upon inoculation.

Immobilization techniques offer a promising strategy for enhancing *rhizobial* survival by entrapping cells within protective matrices. Among the various immobilization approaches, the embedding method [16], whereby cells are encapsulated within hydrogel beads, has attracted considerable attention owing to its operational simplicity, mild processing conditions, and scalability. Sodium alginate (SA), a natural polysaccharide extracted from brown algae, is widely utilized in food science [17], medicine [18], and agriculture [19,20]. SA was selected as the primary matrix material in this study owing to its excellent biocompatibility, biodegradability, and facile gelation in the presence of divalent cations [21]. Specifically, the mild gelation conditions of SA preserve microbial viability during encapsulation, its crosslinking density can be adjusted to tune pore structure [22], and its inherent biodegradability ensures environmental compatibility in soil applications. However, SA-based carriers often suffer from inadequate mechanical strength and excessive swelling in aqueous environments, which can compromise their structural integrity during prolonged storage.

Polyvinyl alcohol (PVA), a synthetic water-soluble polymer, is often combined with SA to improve the mechanical properties and structural stability of composite carriers [23,24,25]. PVA contributes enhanced tensile strength, flexibility, and chemical resistance, effectively offsetting the mechanical limitations of pure SA hydrogels. The SA/PVA blend system thus provides a versatile platform for carrier design, in which the component ratio can be tuned to achieve an optimal balance between biocompatibility and mechanical performance. However, the dense polymeric network of these composite hydrogels can inhibit cell proliferation and impede mass transfer, thereby reducing microbial activity. One promising strategy to overcome this limitation is to incorporate inorganic particles into the hydrogel matrix, with common examples including iron and its oxides [26,27,28], SiO_2_ [29], ZnO [30], and clay minerals [31]. However, these materials do not provide additional nutrients for microbial growth.

Eggshells, as an abundant agricultural waste, are primarily composed of calcium carbonate. Notably, they also contain residual organic matter and trace elements such as zinc, copper, and manganese, which can serve as nutrients for microbial growth and enhance the mechanical properties of the carrier [32,33,34,35]. Therefore, the incorporation of eggshell powder can introduce additional porosity, enhance nutrient exchange, and provide a source of calcium ions that may further stabilize the alginate matrix through ionic crosslinking. Moreover, the utilization of eggshell waste aligns with the principles of sustainable materials engineering by converting a discarded byproduct into a value-added component.

Although SA- or PVA-based carriers have been explored in previous studies [36,37], systematic investigations into the effects of eggshell content, particle size, and SA/PVA ratio on the structural properties, mass transfer characteristics, biodegradability, and long-term storage performance of microorganisms in composite carriers remain lacking. This study aims to investigate the effects of incorporating eggshell powder into SA/PVA composite hydrogels on pore architecture, mass transfer, and *rhizobial* survival. In this work, *rhizobia* were embedded in six eggshell–SA/PVA (E-SA/PVA) composite hydrogel carriers, which were systematically formulated with varying eggshell contents, particle sizes, and SA/PVA ratios. The structural properties, mass transfer capabilities, biodegradability, and storage stability of these carriers under diverse pH and temperature conditions were systematically evaluated. This study offers a practical and sustainable solution for *rhizobial* inoculant preservation.

## 2. Results and Discussion

### 2.1. Evaluation of Hydrogel Carriers

#### 2.1.1. Mass Transfer Properties

**Figure 1:**
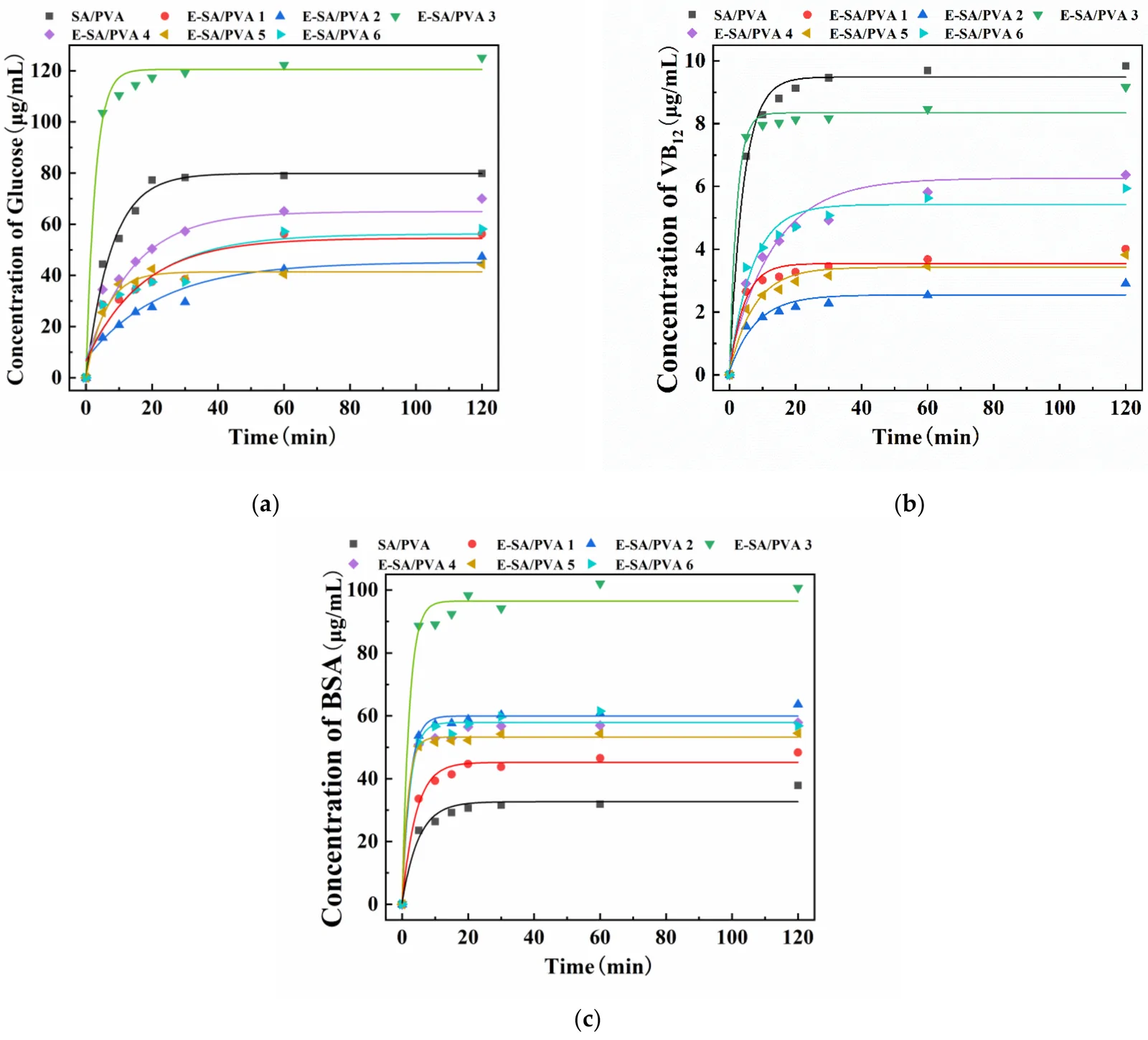
Diffusion curves of glucose (**a**), VB12 (**b**), and BSA (**c**) into water. Data are presented as mean value (*n* = 3).

The mass transfer properties of the E-SA/PVA composite hydrogel carriers were assessed by measuring the diffusion of three probe molecules with distinct molecular weights: glucose, vitamin B12 (VB12), and bovine serum albumin (BSA). The diffusion permeability coefficient (DPC) values of these molecules through the carriers is presented in Table 1 and Figure 1.

E-SA/PVA 3 exhibited the highest DPC value for all three probe molecules, reflecting its exceptionally open pore architecture. The diffusion of glucose through all carriers was relatively rapid, reflecting its small molecular size. VB12, with an intermediate molecular weight, displayed moderate diffusion rates that were sensitive to the pore structure of the carriers. BSA, the largest probe molecule of the three, exhibited the slowest diffusion, with E-SA/PVA 2 and E-SA/PVA 5 maintaining measurably higher diffusion coefficients than the other formulations. These results are consistent with the pore structure analysis, confirming that high porosity and moderate pore sizes synergistically promote efficient molecular transport through the composite carriers.

#### 2.1.2. Mercury Intrusion Porosimetry (MIP) Analysis

**Figure 2:**
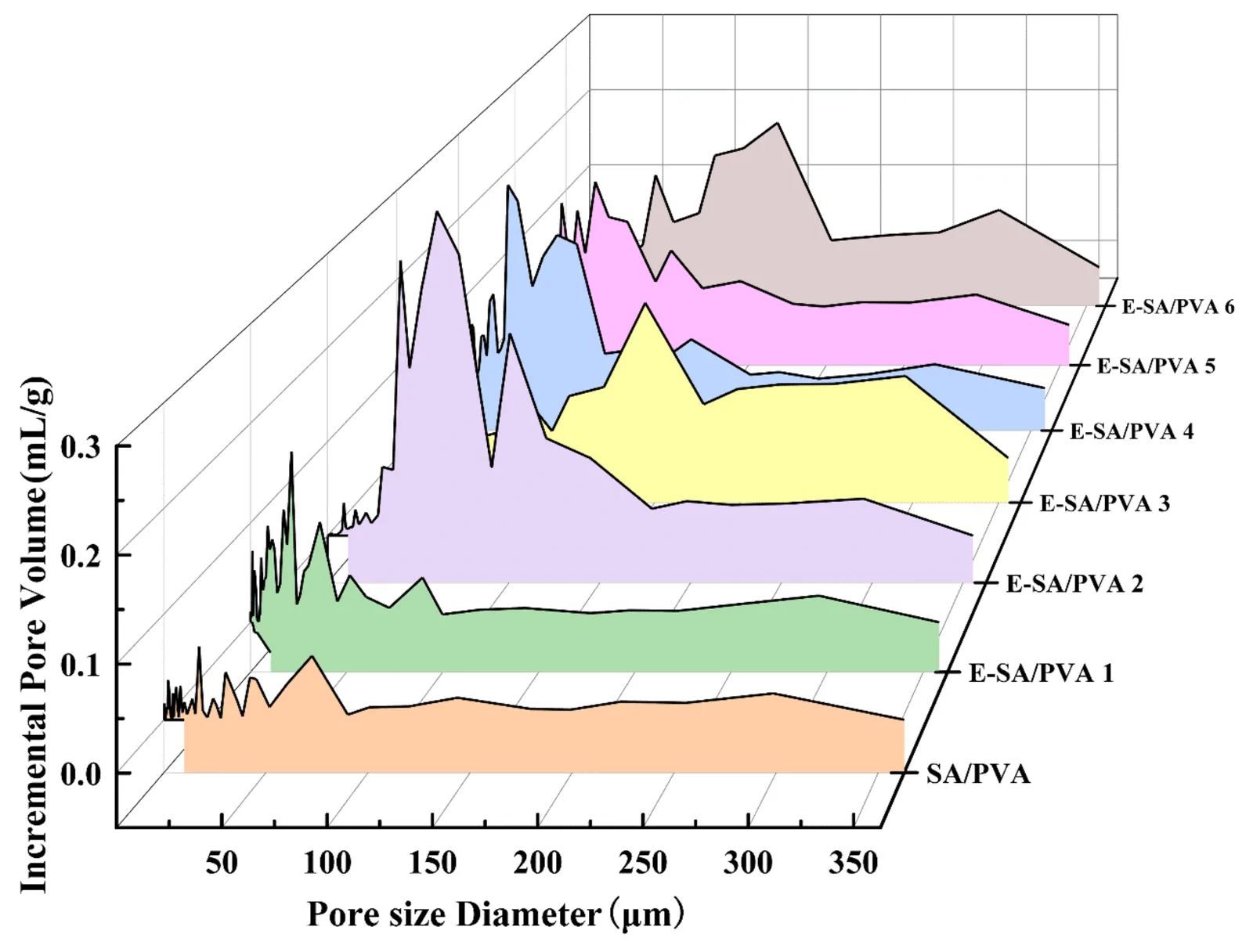
Pore size distribution of SA/PVA and E-SA/PVA 1–6.

The key parameters and pore size distribution data obtained from MIP for SA/PVA and E-SA/PVA 1–6 are summarized in Table 2 and illustrated in Figure 2. According to the data in Table 2, E-SA/PVA 4 exhibited the largest average pore size of 7.84 μm, whereas E-SA/PVA 5 had the smallest average pore size of 2.20 μm. E-SA/PVA 2 exhibited the highest porosity of 80.06%, while E-SA/PVA 3 showed the lowest porosity of 61.17%. In terms of permeability, E-SA/PVA 6 showed the highest value of 32,890.43 mdarcy, and E-SA/PVA 1 had the lowest permeability of 902.81 mdarcy. As shown in Figure 2, the pore size distributions of E-SA/PVA1–6 were mainly concentrated below 20 μm, with the dominant ranges varying across formulations: 0–5, 0–10, 10–15, 0–15, 0–15, and 10–20 μm for E-SA/PVA R1–6, respectively.

E-SA/PVA 2 and E-SA/PVA 5 exhibited the most favorable pore structure characteristics, combining high porosity with moderate average pore sizes and permeability. High porosity facilitates the exchange of nutrients and metabolites, whereas moderate pore sizes provide sufficient surface area for bacterial adhesion while preventing excessive leaching of embedded cells. The observed differences in pore structure among the formulations can be attributed to variations in the SA/PVA ratio and eggshell content, which influence the crosslinking density and the formation of interstitial voids within the composite network.

### 2.2. Characterization of Embedded Rhizobia

#### 2.2.1. FTIR Analysis

**Figure 3:**
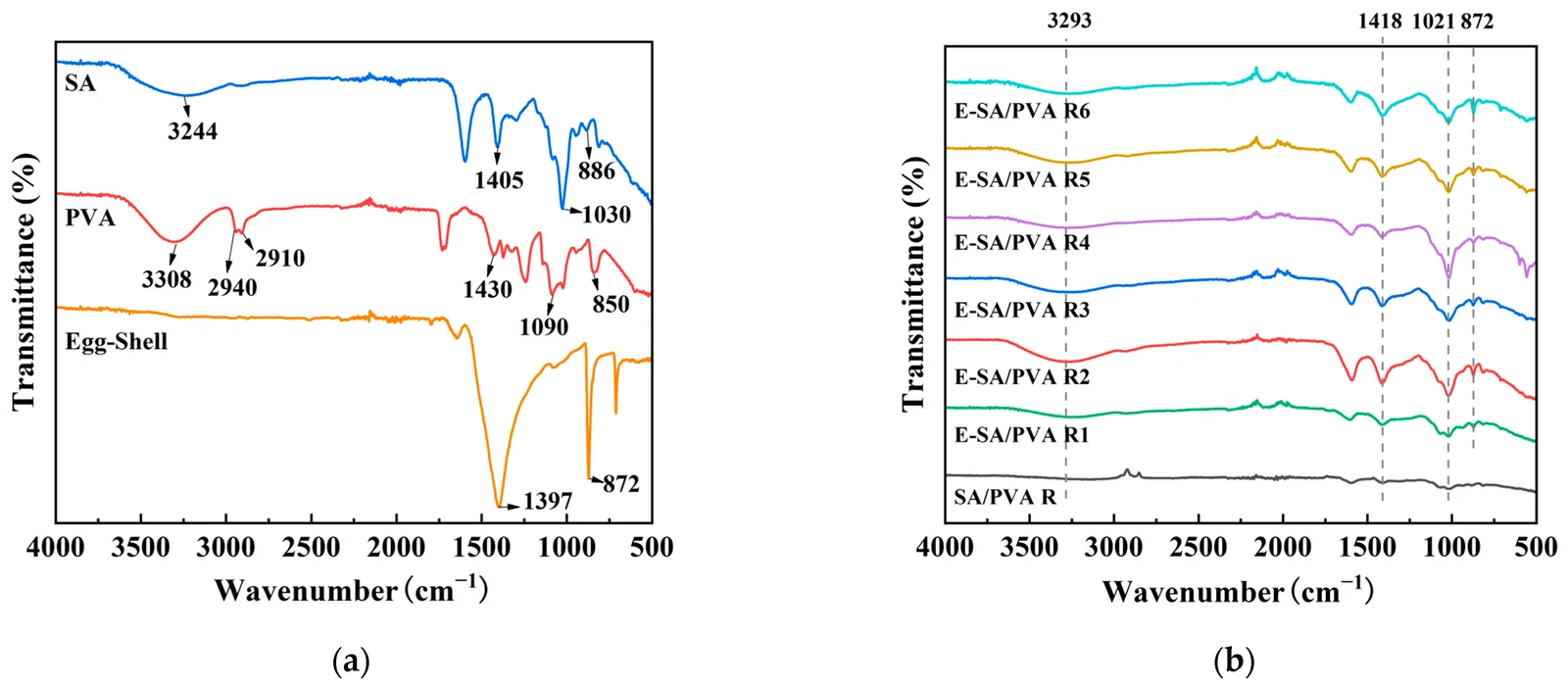
Infrared spectra of raw materials (**a**) and E-SA/PVA R1–R6 (**b**).

The FTIR spectra of the pristine components and the embedded rhizobium agents E-SA/PVA R1–6 are presented in Figure 3. Regarding the individual components, the spectrum of pristine PVA exhibited an O–H stretching vibration at 3308 cm^−1^ [38], C–H stretching and bending vibrations at 2940, 2910, and 1430 cm^−1^, and a C–C stretching band at 850 cm^−1^ [39]. For pristine SA, a broad O–H stretching peak was observed at 3244 cm^−1^, and a C–O stretching band at 1030 cm^−1^ [40]. The eggshell powder displayed characteristic CO_3_^2−^ peaks at 1397 cm^−1^ and 872 cm^−1^ [41].

In the spectra of the E-SA/PVA R1–6 composites, for PVA, the band observed at approximately 823 cm^−1^ was attributed to the B–O stretching vibration from residual B(OH)_4_^−^, indicating the presence of unreacted borate ions in the hydrogel network [42]. Meanwhile, and the O–H stretching peak shifted downward from 3308 cm^−1^ to 3293 cm^−1^, the C–O stretching peak shifted from 1030 cm^−1^ to 1021 cm^−1^, and the COO^−^ symmetric stretching band shifted from 1405 cm^−1^ to 1418 cm^−1^. These spectral shifts are consistent with the reported characteristics of an “egg-box” structure formed between SA and Ca^2+^ ions, indicating potential ionic interactions [43]. The crosslinking is further supported by the insolubility of the formed hydrogels in aqueous media and the enhanced thermal stability observed in the TGA.

For the eggshell powder, the characteristic CO_3_^2−^ peaks were observed in the composites at 872 cm^−1^, confirming that the powder remained on the surface of the carriers. However, the decreased intensity of these peaks compared to pure eggshell powder suggests a partial reaction of CO_3_^2−^ during the boric acid crosslinking process.

#### 2.2.2. Thermogravimetric Analysis

**Figure 4:**
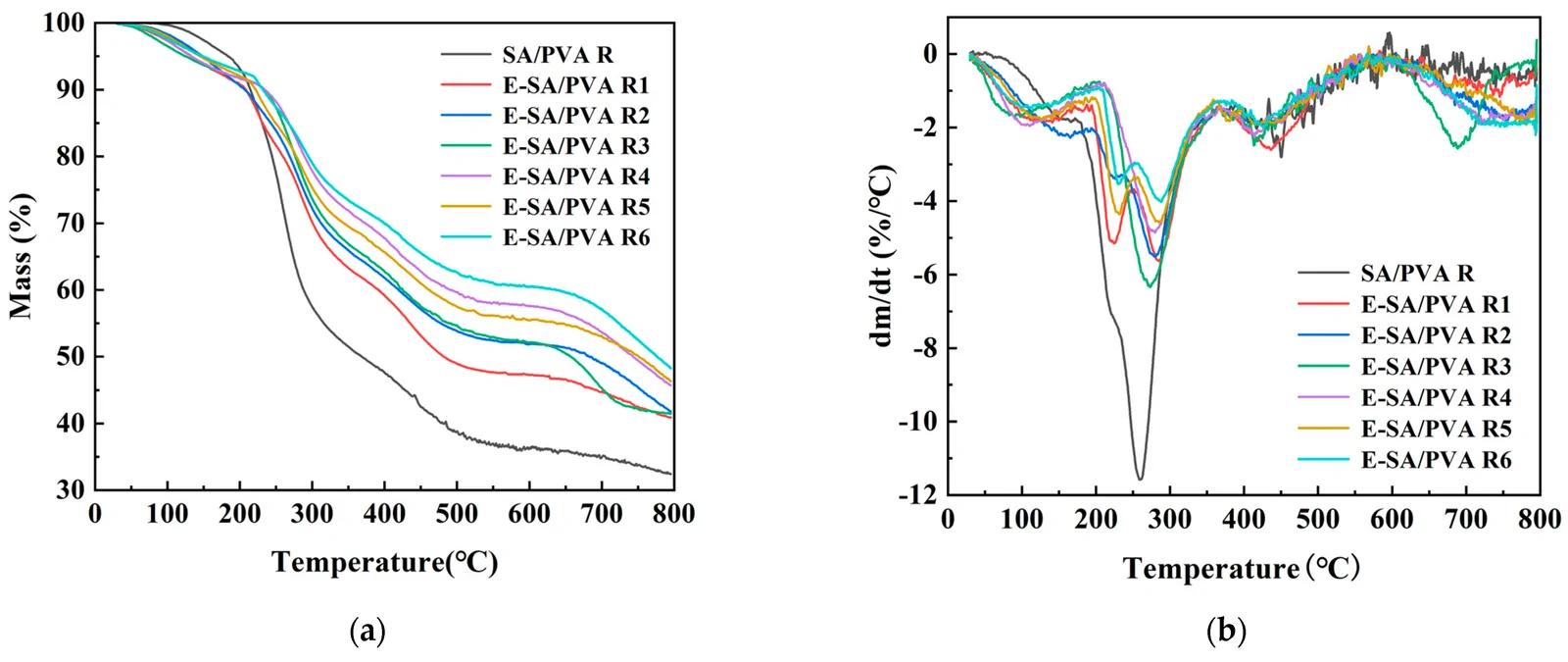
Thermogravimetric curves (**a**) and thermal gravimetric rate curves (**b**) of E-SA/PVA R1–R6.

The thermogravimetric (TG) and derivative thermogravimetric (DTG) results of SA/PVA R and E-SA/PVA R1–6 are shown in Figure 4a,b, with key thermal parameters summarized in Table 3. According to Figure 4a, the temperature at 50% mass loss of SA/PVA R and E-SA/PVA R1–6 was approximately 500 °C. E-SA/PVA R1–6 exhibited a three-stage thermal decomposition profile. The first stage involves moisture loss, occurring in the temperature range of 60–100 °C, during which the mass loss is minimal. The second stage occurs at elevated temperatures, mainly involving the cleavage of polymer chains and protein denaturation. The third stage mainly involves the decomposition of proteins and the breakdown of the polymer backbone, occurring at 400–500 °C. Comparing the DTG curves of SA/PVA R and E-SA/PVA R1–6 in Figure 4b, the thermal weight loss rate of E-SA/PVA R1–6 after adding eggshell was lower than that of SA/PVA R.

As summarized in Table 3, the incorporation of eggshell powder and the subsequent crosslinking significantly altered the thermal decomposition behavior. Compared to the control sample, the onset degradation temperature of the E-SA/PVA R composites was relatively lower. This initial decrease can be attributed to the microstructural changes induced by the eggshell filler. According to the mercury intrusion porosimetry data (Table 2), the addition of eggshell increased the average pore diameter and porosity of the composites. This more porous structure, combined with the polymer-filler interfacial gaps, likely facilitated heat transfer into the material and initiated early chain cleavage at the interfaces. However, the E-SA/PVA R exhibited enhanced high-temperature thermal stability. The 50% mass loss temperature (T_50%_) increased drastically from 368.5 °C to 482.0–777.0 °C, the maximum degradation rate temperature (T_max_) increased from 258.5 °C to 272.5–287.5 °C, and the residual mass increased remarkably from 32.4% to 40.9–48.3%.

Meanwhile, increasing the eggshell addition and decreasing the particle granularity led to higher T50% and residual masses. For example, E-SA/PVA R6 exhibited the best high-temperature stability, with a T50% of 777.0 °C and a residual mass of 48.3%. From a structural perspective, these significant enhancements directly reflect the formation of the crosslinked network. The Ca^2+^ and CO_3_^2−^ from the eggshell participate in forming the “egg-box” structure, which restrict the thermal motion and chain cleavage of the polymers at elevated temperatures. E-SA/PVA R3 exhibited relatively lower thermal parameters compared to its spherical counterpart, E-SA/PVA R2, with the same formulation. This can be attributed to its gel cube shape, where the larger volume hindered the uniform diffusion of crosslinking ions, resulting in a less compact crosslinked network. This structural difference is supported by the MIP data, where R3 exhibited a significantly higher permeability of 25,501.88 mDarcy and a larger average pore diameter of 3.42 μm, compared to 14,054.17 mDarcy and 2.47 μm for R2, indicating the formation of interconnected macropores that facilitate polymer chain decomposition.

#### 2.2.3. SEM Analysis

**Figure 5:**
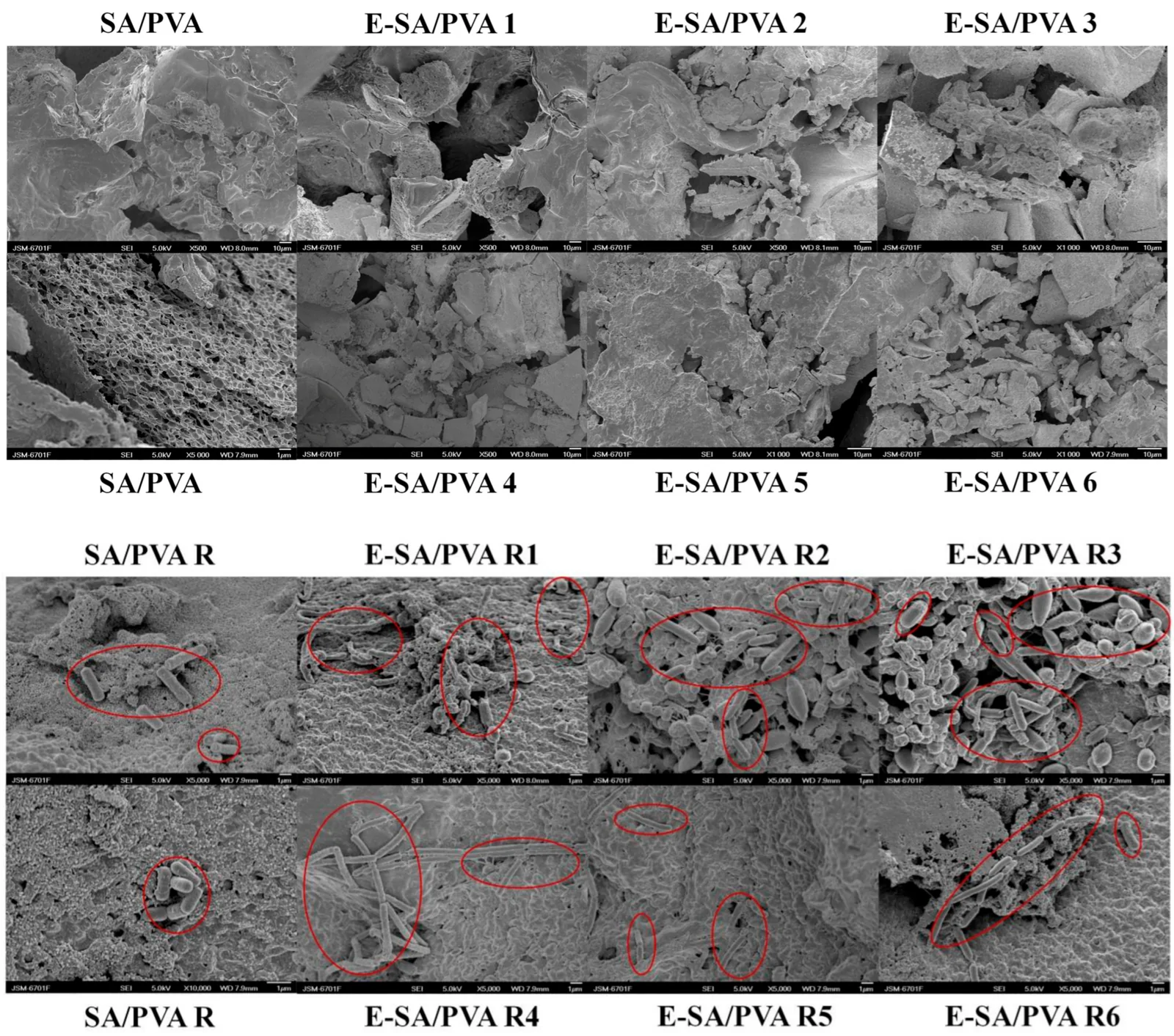
SEM images of SA/PVA, SA/PVA R, E-SA/PVA 1–6 and E-SA/PVA R 1–6. The red circles highlight the rhizobia.

The microstructure and morphology of the composite carriers before and after rhizobia encapsulation were examined by SEM (Figure 5). Prior to encapsulation, the pure SA/PVA carrier exhibited a relatively flat surface with a tightly crosslinked network containing numerous small and dense pores. In contrast, the E-SA/PVA R1–6 carriers displayed a rougher surface with more wrinkles and relatively larger pores. This morphological difference arises because, during the crosslinking process, the added eggshell particles react with the saturated boric acid, altering the network formation and resulting in a looser, more porous structure. The tightly crosslinked network of pure SA/PVA is detrimental to the survival of rhizobia, whereas the wrinkled and porous structure of E-SA/PVA is highly beneficial for the immobilization of rhizobia, as well as the input of nutrients and the output of metabolic products.

Following encapsulation, numerous rod-shaped *rhizobial* cells were observed adhering to the surfaces of the pores and embedded within the polymer matrix across all six formulations, confirming successful encapsulation. The distribution of embedded rhizobia appeared relatively uniform, occupying the surface of the porous network. As shown in the SEM images, a significant number of rhizobia aggregated within E-SA/PVA R2 and R3. This can be attributed to their specific microstructural features. Compared to the spherical beads, the gel cube shape of R3 resulted in a less compact crosslinked network due to the larger volume hindering uniform ion diffusion, thereby forming larger interconnected pores that accommodated more bacteria. Similarly, the specific pore size and permeability of R2 provided a favorable spatial configuration for bacterial aggregation. In comparison, relatively fewer rhizobia were observed on the surfaces of the other formulations, which correlates with their respective pore structures and permeability.

### 2.3. Biodegradability

**Figure 6:**
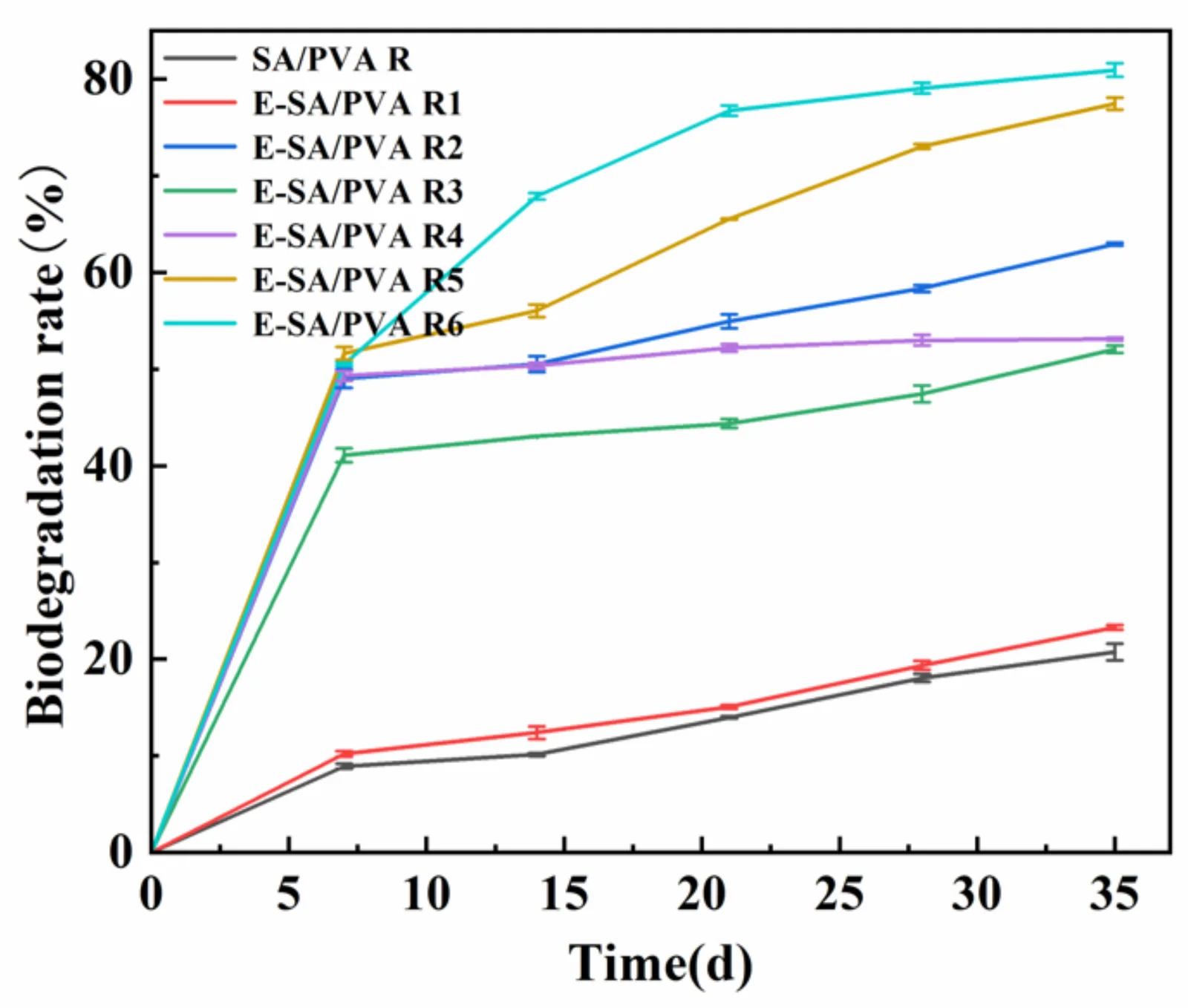
Biodegradation rates of SA/PVA R and E-SA/PVA R 1–6 in soil within 35 days.

The biodegradation rates of the embedded *rhizobial* agents E-SA/PVA 1–6 after 35 days in soil are shown in Figure 6. E-SA/PVA 6 exhibited the highest biodegradation rate, reaching 80.93% at 35 days, which may be attributed to its superior permeability and larger internal pore sizes, facilitating rapid microbial infiltration and participation in the degradation process. Moreover, the relatively high eggshell content and smaller eggshell particle size in E-SA/PVA 6 likely enhanced nutrient availability for microorganisms and promoted microbial enrichment around the sample. E-SA/PVA 5 also showed a relatively high degradation rate of 77.46% at 35 days. The excessively high degradation rates of E-SA/PVA 5 and 6 suggest that these formulations may compromise the controlled release capability, leading to premature release of *rhizobia* into the soil. In contrast, E-SA/PVA 1 exhibited the lowest biodegradation rate throughout the 35-day period, reaching only 23.29% at 35 days, which may be due to its more compact structure, lower permeability, and reduced eggshell content. These characteristics limited both the transport of nutrients required by the embedded *rhizobia* agents and the accumulation of degrading microorganisms on the carrier surface, resulting in a slow release that may delay its effectiveness in practical applications. Among all formulations, E-SA/PVA 2–4 showed moderate biodegradation rates of 62.94%, 52.07%, and 53.13% at 35 days, respectively, demonstrating suitable degradation behavior for achieving sustained release of *rhizobia*.

### 2.4. Storage Stability

#### 2.4.1. Effect of pH on the Number of Viable Rhizobia in Embedded Agents

**Figure 7:**
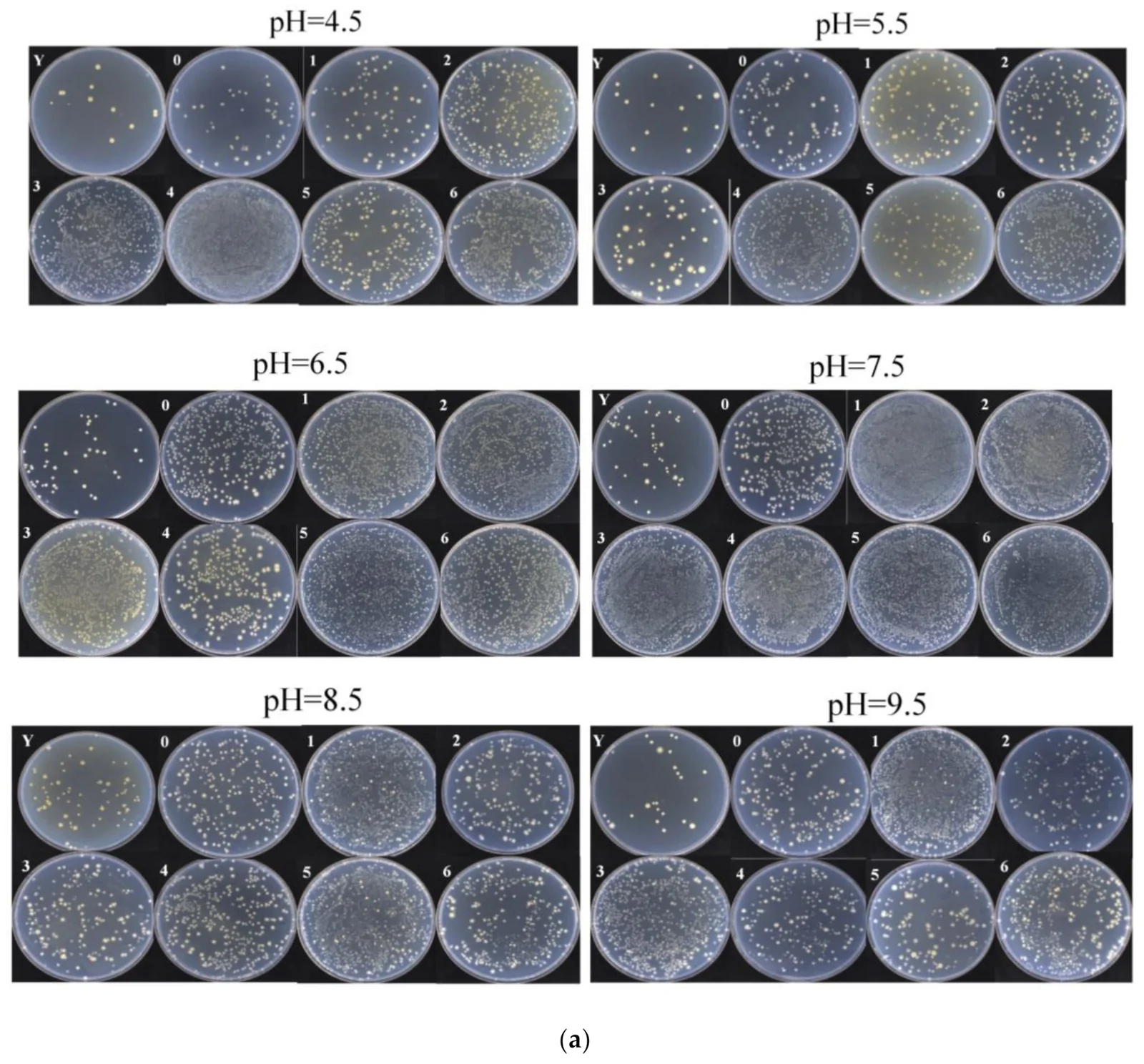

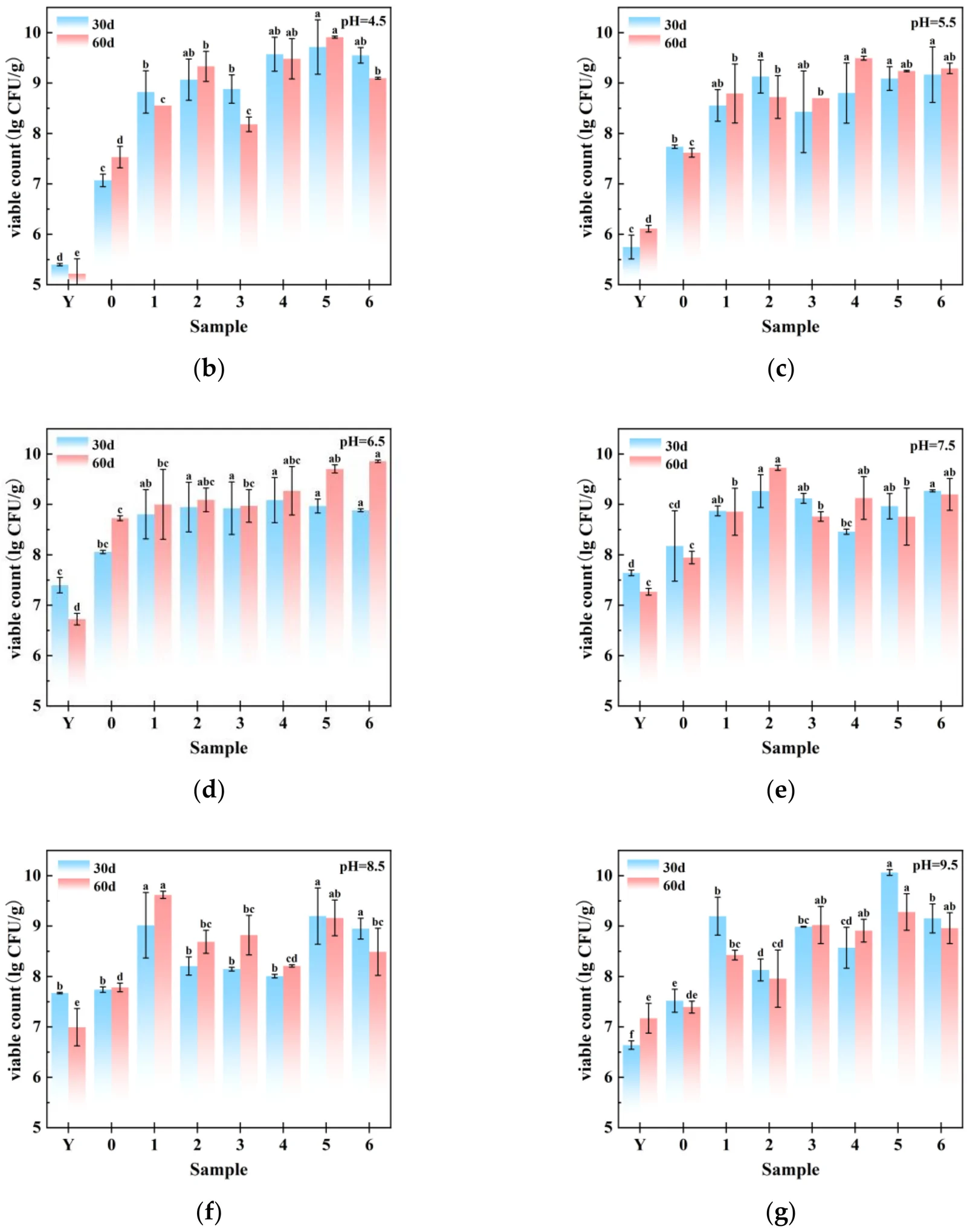
Changes in colony morphology (**a**) and viable bacterial counts at different pH values: (**b**) pH 4.5; (**c**) pH 5.5; (**d**) pH 6.5; (**e**) pH 7.5; (**f**) pH 8.5; (**g**) pH 9.5 of free *rhizobia* (Y), SA/PVA R (0), and E-SA/PVA R1–R6 (1–6) stored for 60 days. Data are presented as mean ± standard deviation (*n* = 3). Different lowercase letters indicate significant differences at *p* < 0.05.

Free *rhizobia* and the embedded *rhizobial* agents E-SA/PVA 1–6 were stored at 28 °C under pH conditions of 4.5, 5.5, 6.5, 7.5, 8.5, and 9.5 for 60 days. The colony morphology was shown in Figure 7a. The viable bacterial counts were determined using the plate counting method at days 30 and 60, and the results are presented in Figure 7b–f. After 60 days of storage, the viable cell counts of all six embedded formulations remained above 8 log CFU/g. In contrast, free *rhizobia* were highly sensitive to pH fluctuations, particularly under extreme pH values. For instance, at pH 4.5, the viable counts of free *rhizobia* decreased to 5.4 log CFU/g at day 30 and 5.22 log CFU/g at day 60, whereas all embedded formulations maintained counts above 9 log CFU/g. These results indicate that the E-SA/PVA composite carriers effectively protected the encapsulated *rhizobia* from environmental stress caused by pH fluctuations, exhibiting excellent biocompatibility. Notably, the survival rates of *rhizobia* under acidic conditions were slightly higher than those under alkaline conditions, suggesting that weakly acidic conditions are optimal for *rhizobial* viability.

Among the embedded formulations, increasing the eggshell content generally enhanced *rhizobial* survival during storage, as evidenced by the higher viable counts in E-SA/PVA 4–6 compared to E-SA/PVA 1–2. However, the cubic-shaped E-SA/PVA 3 exhibited slightly lower viable counts than the spherical E-SA/PVA 2, which may be attributed to the lower permeability and reduced specific surface area of the cubic structure, as well as its inferior mass transfer capability compared to the spherical morphology [44]. Comparison of Figure 7b–g revealed that E-SA/PVA 5 maintained the most stable *rhizobial* survival throughout the storage period, with minimal impact from pH variations. This stability may be attributed to the smaller particle size of the eggshell powder used in E-SA/PVA 5, which increased the contact area with *rhizobia* and facilitated more efficient nutrient uptake from the eggshell components. Therefore, E-SA/PVA 5 is a promising candidate for the stable replenishment *rhizobia* in acidic or alkaline soils over short- to medium-term periods.

#### 2.4.2. Effect of Temperature on the Number of Viable Rhizobia in Embedded Agents

**Figure 8:**
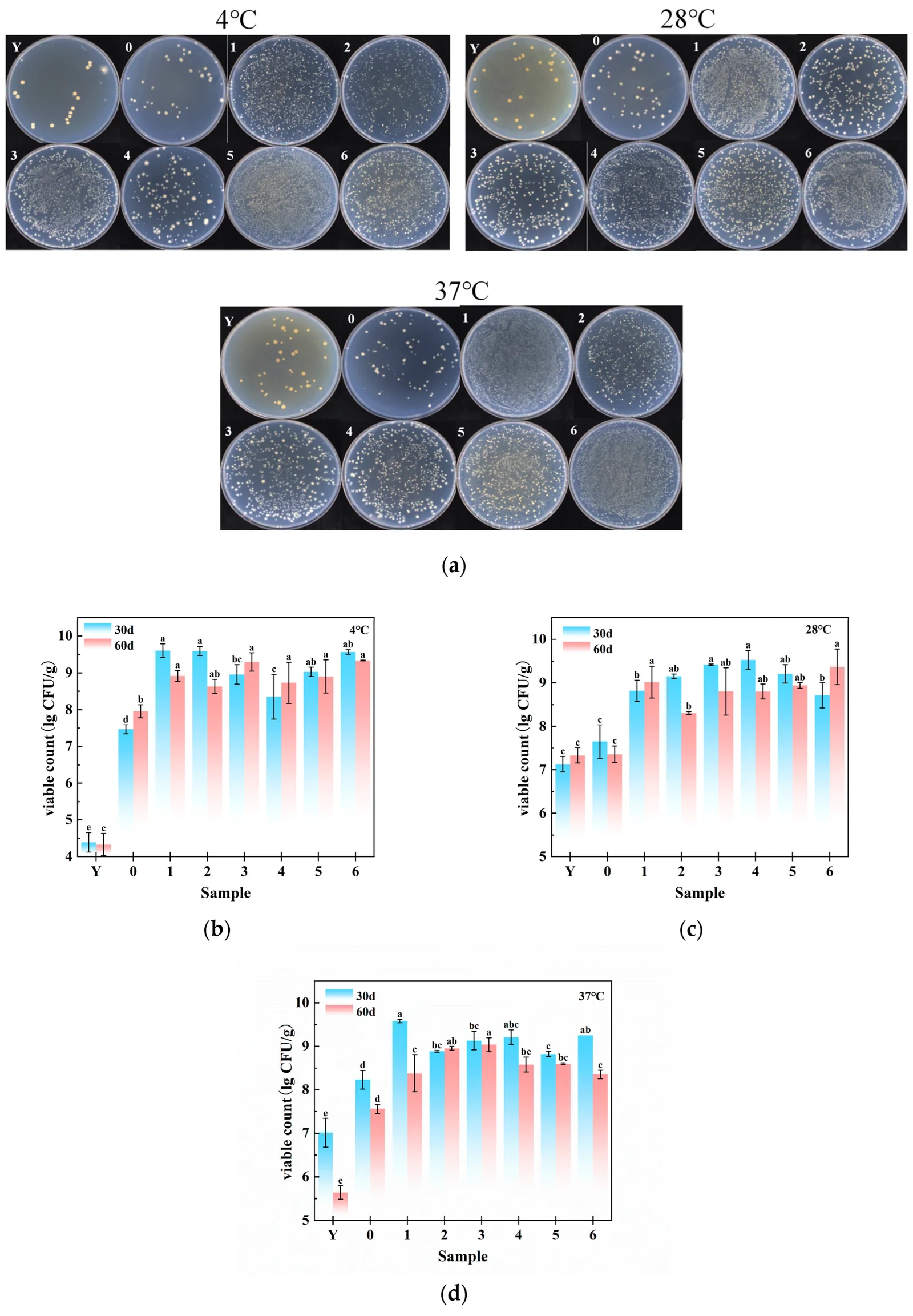
Changes in colony morphology (**a**) and viable bacterial counts at different temperatures: (**b**) 4 °C; (**c**) 28°C; (**d**) 37 °C of free *rhizobia* (Y), SA/PVA R (0), and E-SA/PVA R1–R6 (1–6) stored for 60 days. Data are presented as mean ± standard deviation (*n* = 3). Different lowercase letters indicate significant differences at *p* < 0.05.

Free *rhizobia* and the embedded *rhizobial* agents E-SA/PVA 1–6 were stored at 4 °C, 28 °C, and 37 °C under a constant pH of 7 for 60 days. The viable bacterial counts were determined at days 30 and 60, and the results are presented in Figure 8. Temperature had a highly significant effect on the survival of free *rhizobia*: at 4 °C, the viable counts were 4.39 log CFU/g at day 30 and 4.33 log CFU/g at day 60, while at 37 °C, the corresponding values were 7.01 log CFU/g and 5.64 log CFU/g. In contrast, all six embedded formulations maintained viable counts exceeding 8 log CFU/g after 60 days under all three temperature conditions. These results demonstrate that the E-SA/PVA composite carriers provided significant protection to *rhizobia* against temperature stress, whereas free *rhizobia* were severely inhibited. Therefore, the embedded *rhizobial* agents E-SA/PVA 1–6 can be effectively stored and applied under low-, medium-, and high-temperature conditions without compromising *rhizobial* viability.

#### 2.4.3. Effect of NaCl Concentration on the Number of Viable Rhizobia in Embedded Agents

**Figure 9:**
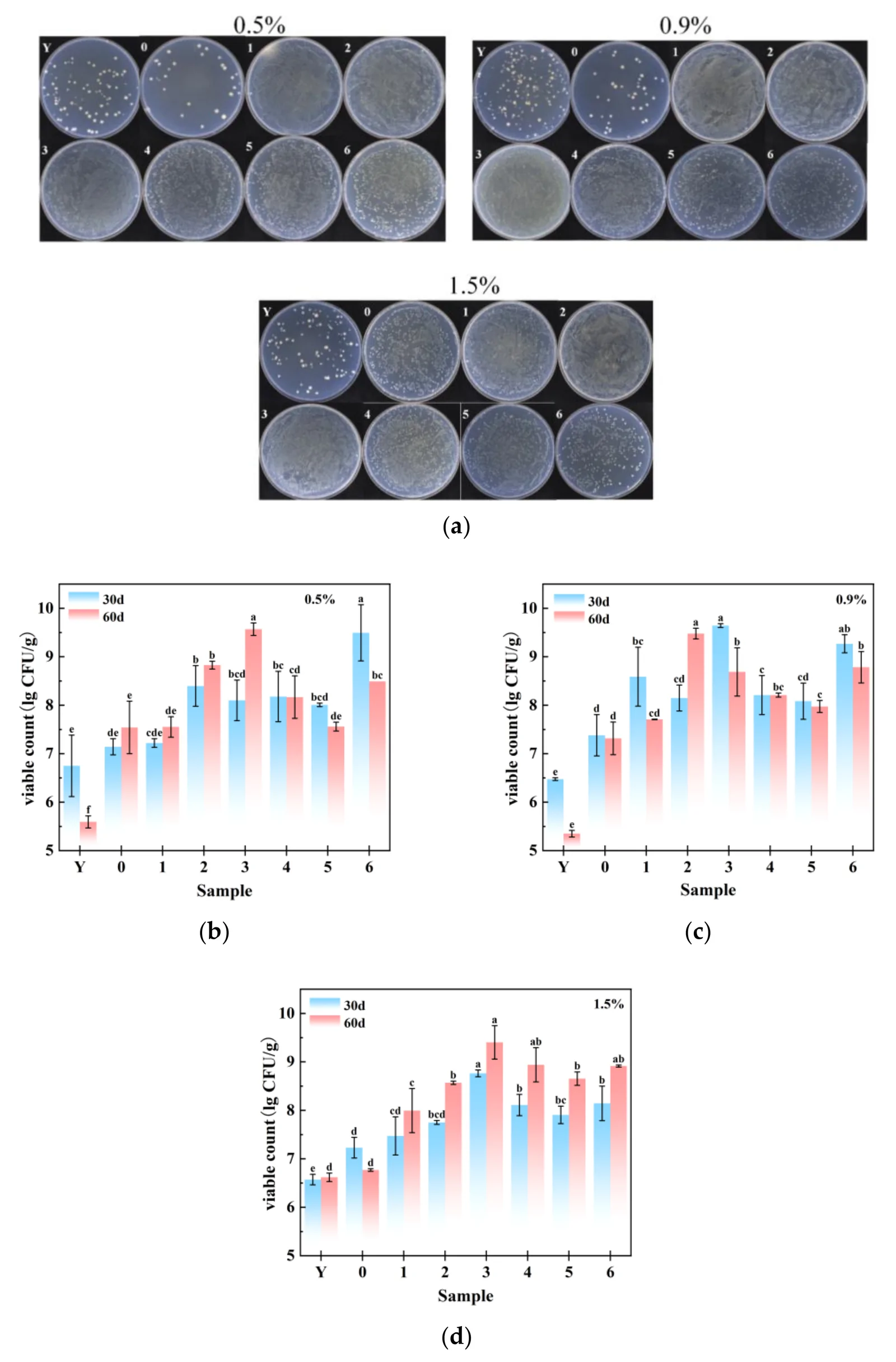
Changes in colony morphology (**a**) and viable bacterial counts at different salt concentrations: (**b**) 0.5%; (**c**) 0.9%; (**d**) 1.5% of free *rhizobia* (Y), SA/PVA R (0), and E-SA/PVA R1–R6 (1–6) stored for 60 days. Data are presented as mean ± standard deviation (*n* = 3). Different lowercase letters indicate significant differences at *p* < 0.05.

The changes in viable bacterial count of free *rhizobia* and embedded *rhizobia* agents SA/PVA R and E-SA/PVA R1–6 stored for 60 days under NaCl concentrations of 0.5%, 0.9%, and 1.5% are shown in Figure 9. The effect of salt concentration on the viability of free *rhizobia* is significant, and their viability is markedly lower than that of embedded *rhizobia* agents under different salt concentration conditions. This can be attributed to the fact that elevated NaCl concentrations induce osmotic stress, causing cellular dehydration and plasmolysis in free rhizobia, while also disrupting membrane integrity and enzyme activity through ion toxicity [45]. In contrast, the number of viable bacteria in SA/PVA R was higher than that in the free *rhizobia*, but lower than that in the embedded *rhizobial* agents E-SA/PVA R1–6. This difference is primarily attributable to the absence of eggshells in SA/PVA R; without eggshell-derived Ca^2+^, the matrix lacked the additional membrane-stabilizing effect provided by calcium ions, despite its denser gel network structure. The hydrogel matrix of the embedded carriers acts as a physical barrier that buffers osmotic shock and moderates the influx of Na^+^ ions, thereby protecting the encapsulated rhizobia from direct salt exposure [46]. Furthermore, the Ca^2+^ ions released from eggshell powder may help stabilize bacterial cell membranes under saline conditions, as divalent cations, particularly Ca^2+^, play a crucial role in crosslinking lipopolysaccharide molecules on the outer membrane of Gram-negative bacteria, thereby maintaining membrane integrity under osmotic stress [47].

Under different salt concentrations, the survival status of *rhizobia* in the embedded *rhizobial* agents E-SA/PVA R2–4 and E-SA/PVA R6 was relatively stable. After 60 days, the survival of *rhizobia* exceeded 8 log CFU/g, meeting the requirements of microbial fertilizer standards. Experiments have shown that both high and low salt concentrations have inhibitory effects on the survival of *rhizobia*, but the embedded *rhizobia* agents E-SA/PVA R1–R6 exhibit protective effects on *rhizobia* under different salt concentrations. Overall, embedded *rhizobial* agents E-SA/PVA R2–4 and E-SA/PVA R6 can be used as microbial fertilizers in salt stressed soils.

## 3. Conclusions

In this study, six eggshell–sodium alginate/polyvinyl alcohol (E-SA/PVA) composite carriers with varying component ratios were prepared for *rhizobia* encapsulation. FTIR and thermogravimetric analyses confirmed the effective crosslinking between SA and PVA and enhanced thermal stability conferred by eggshell incorporation. Mercury intrusion porosimetry and mass transfer experiments identified E-SA/PVA 2 and E-SA/PVA 5 as possessing the most favorable pore structure and diffusion properties, which was further supported by SEM observations confirming successful *rhizobia* encapsulation across all formulations. Biodegradation tests revealed that E-SA/PVA 2–4 exhibited moderate degradation rates ranging from 52.07% to 62.94% at 35 days, ensuring a balance between structural integrity and controlled release. After 60 days of storage under various pH and temperature conditions, all formulations maintained viable counts above 8 log CFU/g, with the composite carriers providing effective protection against adverse conditions that severely affected free *rhizobia*. These carriers offer a scalable and cost-effective strategy for *rhizobial* inoculant formulation, with potential applications in soil amendment. While current results have demonstrated the protective efficacy of the composite hydrogel carriers, longer-term stability validation and the evaluation of practical plant growth effects will be addressed in future studies.

## 4. Materials and Methods

### 4.1. Materials

SA (AR, 90%), PVA (η = 45.0–55.0 mPa·s) were purchased from Macklin Biochemical Co., Ltd. (Shanghai, China). Eggshells were collected from local markets and pretreated as described below. *Rhizobacteria* (GAU00124) were provided by the research group of Professor Yao from Gansu Agricultural University. All other reagents were of analytical grade and used as received without further purification.

### 4.2. Preparation of Embedded Rhizobia Agents

**Figure 10:**
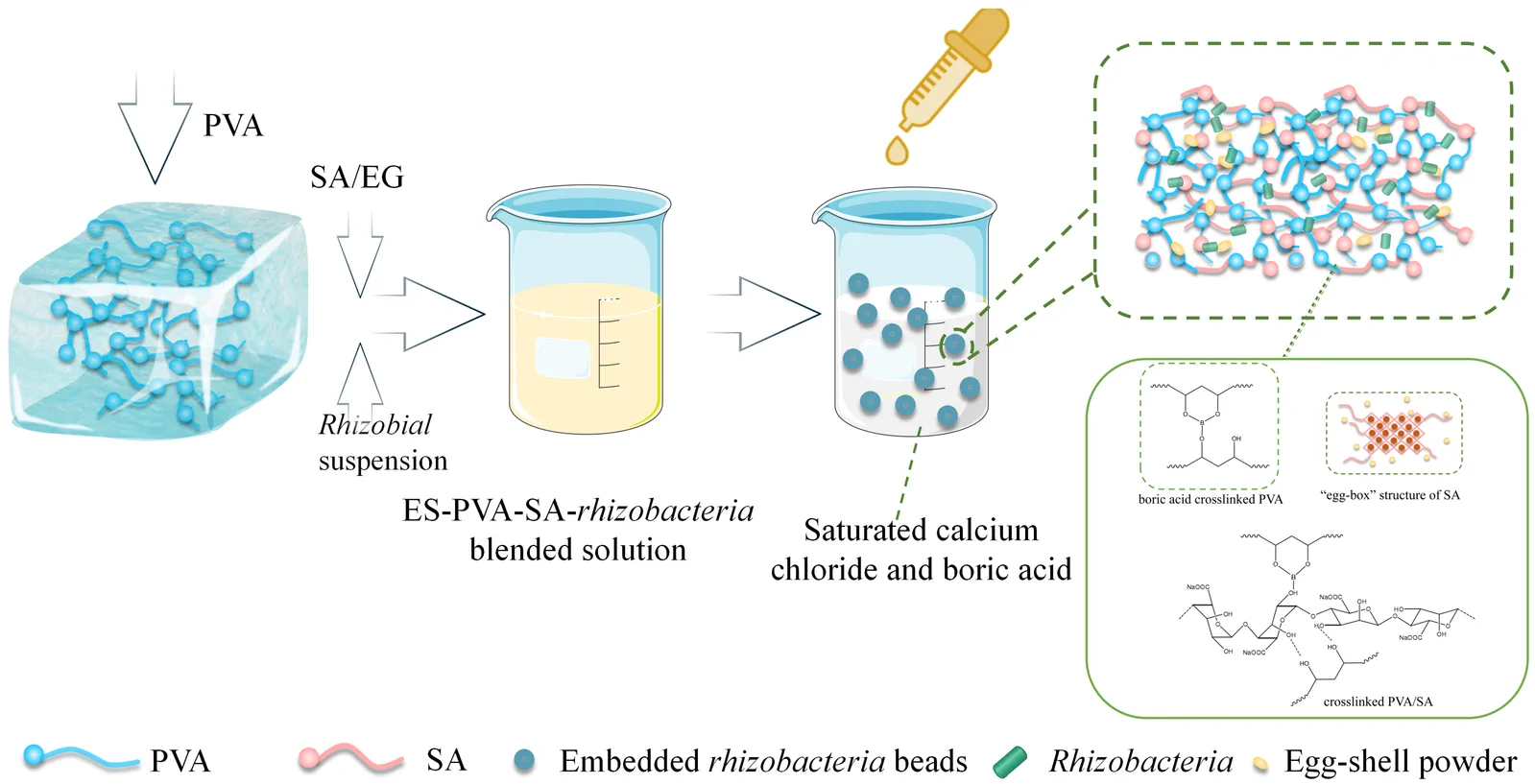
Schematic illustration of the preparation of E-SA/PVA embedded *rhizobia* beads.

Eggshells were dried at 80 °C for 12 h, then ground and sieved to obtain powders of 200 and 320 mesh. A single *rhizobial* colony was picked from a yeast mannitol agar (YMA) plate using an inoculating loop and transferred into 80 mL of sterilized YMA broth [48]. The inoculated broth was incubated at 28 °C for 72 h to obtain a bacterial suspension with a concentration of approximately 10^8^ CFU/mL, as determined by the plate counting method. The suspension was then centrifuged twice at 8000 rpm, the supernatant discarded, and the pellet resuspended in sterile water to a final volume of 10 mL, yielding a concentrated suspension of approximately 10^9^ CFU/mL for subsequent encapsulation. Six different formulations for the encapsulation matrix were prepared, designated as E-SA/PVA R1 through E-SA/PVA R6, with their compositions detailed in Table 4. Based on our previous study [49], 1 g of SA, 1.5 g of PVA, and a specified amount of eggshell powder for each formulation were sterilized under UV light for 1 h. The sterilized powders were gradually added into 90 mL of sterile water in a three-necked flask under continuous stirring, followed by heating in a 50 °C water bath for 30 min to ensure complete dissolution. Then, 10 mL of the concentrated *rhizobial* suspension was added to the polymer solution and stirred for an additional 30 min to achieve homogeneous mixing. The resulting mixture was fabricated into two shapes: spherical beads and gel cubes. As shown in Figure 10, spherical beads were formed by dropping the mixture through a plastic pipette into a crosslinking solution containing saturated calcium chloride and boric acid. Gel cubes were formed by slowly adding the crosslinking solution into the polymer mixture under continuous stirring to yield a bulk gel. After 2 h of crosslinking, all samples were removed, rinsed twice with sterile water, freeze-dried, and sealed for subsequent use. For the evaluation of mass transfer and other properties, blank hydrogel carriers were prepared as described above, using the same formulations listed in Table 4 but without the addition of *rhizobia*. These blank carriers were denoted as SA/PVA and E-SA/PVA 1–6.

### 4.3. Mass Transfer Performance Evaluation of Hydrogel Carriers

Glucose (M*_W_* 180 Da), vitamin B12 (M*_W_* 1355 Da), and bovine serum albumin (M*_W_* 66 kDa) were selected as probe molecules. The procedure was as follows: Each hydrogel carrier sample (SA/PVA and E-SA/PVA 1–6) was pre-weighed and then immersed in the solution of probe molecules for 72 h to reach adsorption equilibrium, with the solution refreshed every 24 h. Each sample was then transferred to 20 mL of deionized water and shaken at 180 rpm, 28 °C. Aliquots (1 mL) were collected at 5, 10, 15, 20, 30, 60, and 120 min, diluted with an equal volume of deionized water, and analyzed for the concentrations of the probe molecules. The diffusion permeability coefficient (*DPC*) was derived from Fick’s second law of diffusion [50]:

(1) $$DPC=\frac{V_{1}V_{2}}{A(V_{1}{+V}_{2})}K$$

where *V*_1_ is the volume of the oscillation solution (m^3^), *V*_2_ is the total volume of the gel beads (m^3^), *A* is the total surface area of the beads (m^2^), and *K* is the slope of the linear plot of ln[(Cf − C0)/(Cf − Ct)] versus time t. C0, Ct and Cf denote the solute concentrations in the bulk solution at the initial time, time t, and equilibrium, respectively.

To elucidate the structural basis for the observed mass transfer behaviors, the pore size distribution, average pore size, porosity, and permeability of the composites were determined by mercury intrusion porosimetry (MicroActive AutoPore V 9600, Micromeritics Instrument Corporation, Norcross, GA, USA) over a pressure range of 0.10–61,000 Pa.

### 4.4. Characterization of Embedded Rhizobia Agents

The characteristic functional groups of SA, PVA, eggshell powder, and E-SA/PVA R1–R6 were analyzed by Fourier-transform infrared spectroscopy (FTIR-650, Gangdong Science & Technology Co., Ltd., Tianjin, China) over a wavenumber range of 500–4000 cm^−1^ at a resolution of 4 cm^−1^. The surface morphology of the embedded samples was examined using a field emission scanning electron microscope (JSM-6701F, JEOL Ltd., Tokyo, Japan). Prior to observation, the samples were fixed with 2.5% glutaraldehyde at 4 °C for 4 h, sequentially dehydrated with graded ethanol solutions, immersed in tert-butanol, and sputter-coated with gold. The thermal degradation behavior of the samples was investigated using a simultaneous thermal analyzer (TG 209 F3, NETZSCH-Gerätebau GmbH, Selb, Germany) from 30 to 800 °C at a heating rate of 10 °C/min under a nitrogen atmosphere, 50 mL/min.

### 4.5. Determination of Biodegradation Rate

Three replicates of each E-SA/PVA R1–R6 sample were weighed and buried approximately 5 cm below the soil surface in pots. Every three days, 100 mL of distilled water was evenly added to the pots to maintain soil moisture. The samples were retrieved at intervals of 0, 7, 14, 21, 28, and 35 days, respectively, and subsequently dried thoroughly in an oven to remove residual moisture.

The biodegradation rate (*BR*) of each sample was calculated using formula (2) [51]:

(2) $$BR=\frac{m_{0}-m_{1}}{m_{0}}\times 100\%$$

where *m*_0_ is the initial weight (g) of each sample before burial, and *m*_1_ is the weight (g) after drying at each time point.

### 4.6. Evaluation of Storage Stability

To evaluate the protective effects of the composite carriers on *rhizobia* under various environmental conditions, the impacts of pH and temperature on *rhizobial* viability were investigated over a 60-day storage period. For the pH-dependent study, the incubation temperature was maintained at 28 °C, and the pH values were adjusted to 4.5, 5.5, 6.5, 7.5, 8.5, and 9.5. For the temperature-dependent study, the pH was set to 7.0, and the incubation temperatures were 4 °C, 28 °C, and 37 °C. The NaCl concentrations were set at 0.5%, 0.9%, and 1.5%, respectively.

Approximately 0.5 g of each embedded *rhizobia* agents E-SA/PVA 1–6 was weighed and placed into culture vessels, which were then incubated in a controlled environment for up to 60 days. Samples were retrieved at day 30 and day 60, and the viable *rhizobia* counts in each carrier were determined using the plate counting method. The number of viable *rhizobia* was calculated using formula (3) [49]:

(3) $$n_{m}=\frac{{kV}_{1}\bar{X}}{V_{2}m_{0}}\times 10^{-8}$$

where *n_m_* is the number of viable *rhizobia* per gram of sample (CFU/g); $\bar{X}$ is the average colony count from plate counts (colony-forming units, CFUs); *k* is the dilution factor; *V*_1_ is the volume of sterile water added during shaking (mL); *m*_0_ is the mass of the sample (g); *V*_2_ is the volume of *rhizobial* suspension used for plating (mL).

### 4.7. Statistical Analysis

All experiments were performed in triplicate (*n* = 3), and the results are expressed as the mean ± standard deviation (SD). SPSS 16.0 (One-Way ANOVA) was used for significance analysis (*p* < 0.05), and the results were represented by superscript.

## Figures and Tables

**Table 1:** DPC values of SA/PVA and E-SA/PVA 1–6.

Sample	SA/PVA	E-SA/PVA 1	E-SA/PVA 2	E-SA/PVA 3	E-SA/PVA 4	E-SA/PVA 5	E-SA/PVA 6
DPC for glucose (×10^−11^ m^2^/min)	3.387	3.561	1.769	11.73	2.394	1.551	3.785
DPC for VB12 (×10^−11^ m^2^/min)	2.741	1.555	1.475	6.468	2.137	1.637	2.669
DPC for BSA (×10^−11^ m^2^/min)	1.038	2.517	1.831	12.86	3.243	3.861	2.722

**Table 2:** Key data from mercury intrusion porosimetry for SA/PVA and E-SA/PVA 1–6.

Sample	Total Pore Area (m^2^/g)	Average Pore Diameter (μm)	Porosity (%)	Permeability (Mdarcy)
SA/PVA	1.9	1.96	57.49	14,944.53
E-SA/PVA 1	3.26	2.29	71.96	902.81
E-SA/PVA 2	0.24	2.47	80.06	14,054.17
E-SA/PVA 3	0.17	3.42	61.17	25,501.88
E-SA/PVA 4	1.16	7.84	79.66	5426.3
E-SA/PVA 5	0.27	2.2	73.35	12,121.42
E-SA/PVA 6	0.84	6.02	67.82	32,890.43

**Table 3:** The thermal parameters of E-SA/PVA 1–6.

Sample	Onset Degradation Temperature (°C)	50% Mass Loss Temperature (T_50%_, °C)	Maximum Degradation Rate Temperature (T_max_, °C)	Residual Mass (%)
SA/PVA R	184.0	368.5	258.5	32.4
E-SA/PVA R1	133.5	482.0	285.5	40.9
E-SA/PVA R2	149.0	683.0	278.0	41.8
E-SA/PVA R3	126.0	657.0	272.5	41.5
E-SA/PVA R4	133.0	744.0	278.0	45.7
E-SA/PVA R5	146.0	751.0	284.5	46.3
E-SA/PVA R6	149.0	777.0	287.5	48.3

**Table 4:** Formulations of the embedded matrix.

Sample	SA (g/100 mL)	PVA (g/100 mL)	Eggshell Granularity (Mesh Number)	The Amount of Eggshell Added (%)	Shape
SA/PVA R	1	1.5	-	-	Spherical beads
E-SA/PVA R1	1	1.5	200	20	Spherical beads
E-SA/PVA R2	1	1.5	200	30	Spherical beads
E-SA/PVA R3	1	1.5	200	30	Gel cubes
E-SA/PVA R4	1	1.5	200	40	Spherical beads
E-SA/PVA R5	1	1.5	320	30	Spherical beads
E-SA/PVA R6	1	1.5	320	40	Spherical beads

## Data Availability

The data presented in this study are available on request from the corresponding author.

## Abbreviations

The following abbreviations are used in this manuscript:

FTIR	Fourier-transform infrared
TGA	Thermogravimetric analysis
SEM	Scanning electron microscopy
E-SA/PVA	Eggshell–sodium alginate/polyvinyl alcohol
CFU	Colony-forming unit
PGPR	Plant growth-promoting *rhizobacteria*
SA	Sodium alginate
PVA	Polyvinyl alcohol
VB12	Vitamin B12
BSA	Bovine serum albumin
DPC	Diffusion permeability coefficient
MIP	Mercury intrusion porosimetry
TG	Thermogravimetric
DTG	Derivative thermogravimetric
AR	Analytical reagent
YMA	Yeast mannitol agar
UV	Ultraviolet
MW	Molecular weight
Da	Dalton
kDa	Kilodalton
BR	Biodegradation rate
